# Breast cancer patient delay in Fukushima, Japan following the 2011 triple disaster: a long-term retrospective study

**DOI:** 10.1186/s12885-017-3412-4

**Published:** 2017-06-19

**Authors:** Akihiko Ozaki, Shuhei Nomura, Claire Leppold, Masaharu Tsubokura, Tetsuya Tanimoto, Takeru Yokota, Shigehira Saji, Toyoaki Sawano, Manabu Tsukada, Tomohiro Morita, Sae Ochi, Shigeaki Kato, Masahiro Kami, Tsuyoshi Nemoto, Yukio Kanazawa, Hiromichi Ohira

**Affiliations:** 1 Department of Surgery, Minamisoma Municipal General Hospital, 2-54-6 Takamicho, Haramachi, Minamisoma, Fukushima, 975-0033 Japan; 2 Department of Epidemiology and Biostatistics, School of Public Health, Imperial College London, London, SW7 2AZ UK; 3 Department of Research, Minamisoma Municipal General Hospital, Minamisoma, Fukushima, 975-0033 Japan; 4 Department of Radiation Protection, Minamisoma Municipal General Hospital, Minamisoma, Fukushima, 975-0033 Japan; 5 Department of Internal Medicine, Jyoban Hospital of Tokiwa Foundation, Iwaki, Fukushima, 972-8322 Japan; 6 Department of Medical Oncology, Fukushima Medical University, Fukushima, 960-1295 Japan; 7 Department of Internal Medicine, Soma Central Hospital, Soma, Fukushima, 976-0016 Japan; 8 Research Institute of Innovative Medicine, Jyoban Hospital of Tokiwakai Group, Iwaki, Fukushima, 972-8322 Japan; 9 Medical Governance Research Institute, Minato-ku, Tokyo, 108-0074 Japan; 10 Department of Home Medical Care, Minamisoma Municipal General Hospital, Minamisoma, Fukushima, 975-0033 Japan; 11 Department of Gastroenterology, Minamisoma Municipal General Hospital, Minamisoma, Fukushima, 975-0033 Japan; 12 Department of Epidemiology and Biostatistics, Teikyo University Graduate School of Public Health, Minamisoma, Tokyo, 173-8605 Japan; 13 Department of Global Health Policy, Graduate School of Medicine, The University of Tokyo, Minamisoma, Tokyo, 113-0033 Japan

**Keywords:** Breast cancer, Patient delay, Social support, Psychosocial stress, Health service, Fukushima, Minamisoma, Nuclear power plant, Disaster

## Abstract

**Background:**

Little information is available concerning how patient delay may be affected by mass disasters. The main objectives of the present study are to identify whether there was a post-disaster increase in the risk of experiencing patient delay among breast cancer patients in an area affected by the 2011 triple disaster in Fukushima, Japan, and to elucidate factors associated with post-disaster patient delay. Sociodemographic factors (age, employment status, cohabitant status and evacuation status), health characteristics, and health access- and disaster-related factors were specifically considered.

**Methods:**

Records of symptomatic breast cancer patients diagnosed from 2005 to 2016 were retrospectively reviewed to calculate risk ratios (RRs) for patient delay in every year post-disaster compared with the pre-disaster baseline. Total and excessive patient delays were respectively defined as three months or more and twelve months or more from symptom recognition to first medical consultation. Logistic regression analysis was conducted for pre- and post-disaster patient delay in order to reveal any factors potentially associated with patient delay, and changes after the disaster.

**Results:**

Two hundred nineteen breast cancer patients (122 pre-disaster and 97 post-disaster) were included. After adjustments for age, significant post-disaster increases in RRs of experiencing both total (RR: 1.66, 95% Confidence Interval (CI): 1.02–2.70, *p* < 0.05) and excessive patient delay (RR: 4.49, 95% CI: 1.73–11.65, *p* < 0.01) were observed. The RRs for total patient delay peaked in the fourth year post-disaster, and significant increases in the risk of excessive patient delay were observed in the second, fourth, and fifth years post-disaster, with more than five times the risk observed pre-disaster. A family history of any cancer was the only factor significantly associated with total patient delay post-disaster (odds ratio: 0.38, 95% CI: 0.15–0.95, *p* < 0.05), while there were no variables associated with delay pre-disaster.

**Conclusions:**

The triple disaster in Fukushima appears to have led to an increased risk of patient delay among breast cancer patients, and this trend has continued for five years following the disaster.

**Electronic supplementary material:**

The online version of this article (doi:10.1186/s12885-017-3412-4) contains supplementary material, which is available to authorized users.

## Background

Breast cancer is the most common cancer and cause of cancer death for females, making it a significant part of the global cancer burden [1]. A considerable proportion of patients seek medical consultation only after they notice symptoms, such as a breast lump [2]. Among these symptomatic patients, patient delay, generally defined as a delay in first medical consultation of three months or longer following the first recognition of symptoms [3, 4], is a problem that may lead to a late stage diagnosis and worsened prognosis of breast cancer [5, 6]. Associations have been found between clinical, sociodemographic, and psychosocial factors and patient delay [4, 7–9]. Yet, in contrast to a predominant research focus on associations between individual characteristics and patient delay, there is still limited understanding of the broader societal factors that may influence patient delay.

Recently, studies have underlined the importance of the context in which patients discover their symptoms and seek help. For instance, patients who are socially isolated or feel overwhelming responsibilities and stress concerning work or care of family members may be at higher risk of delaying medical consultation [2, 4, 7, 8]. In addition, poor access to medical services may contribute to the delays [4, 8]. The above literature highlights the importance of accounting for the social contexts patients inhabit, in order to fully understand the causes of patient delay. However, this is a difficult point to accomplish given that social contexts can change over time; previous studies have not addressed potential relationships between these changes and patient delay [4, 5, 8, 9]. Mass disasters provide a unique opportunity to assess how rapidly-changing social contexts may impact patient delay, as they can simultaneously disintegrate social connections of victims and access to medical institutions, while exposing disaster victims to high levels of stress [10–13].

**Fig. 1:**
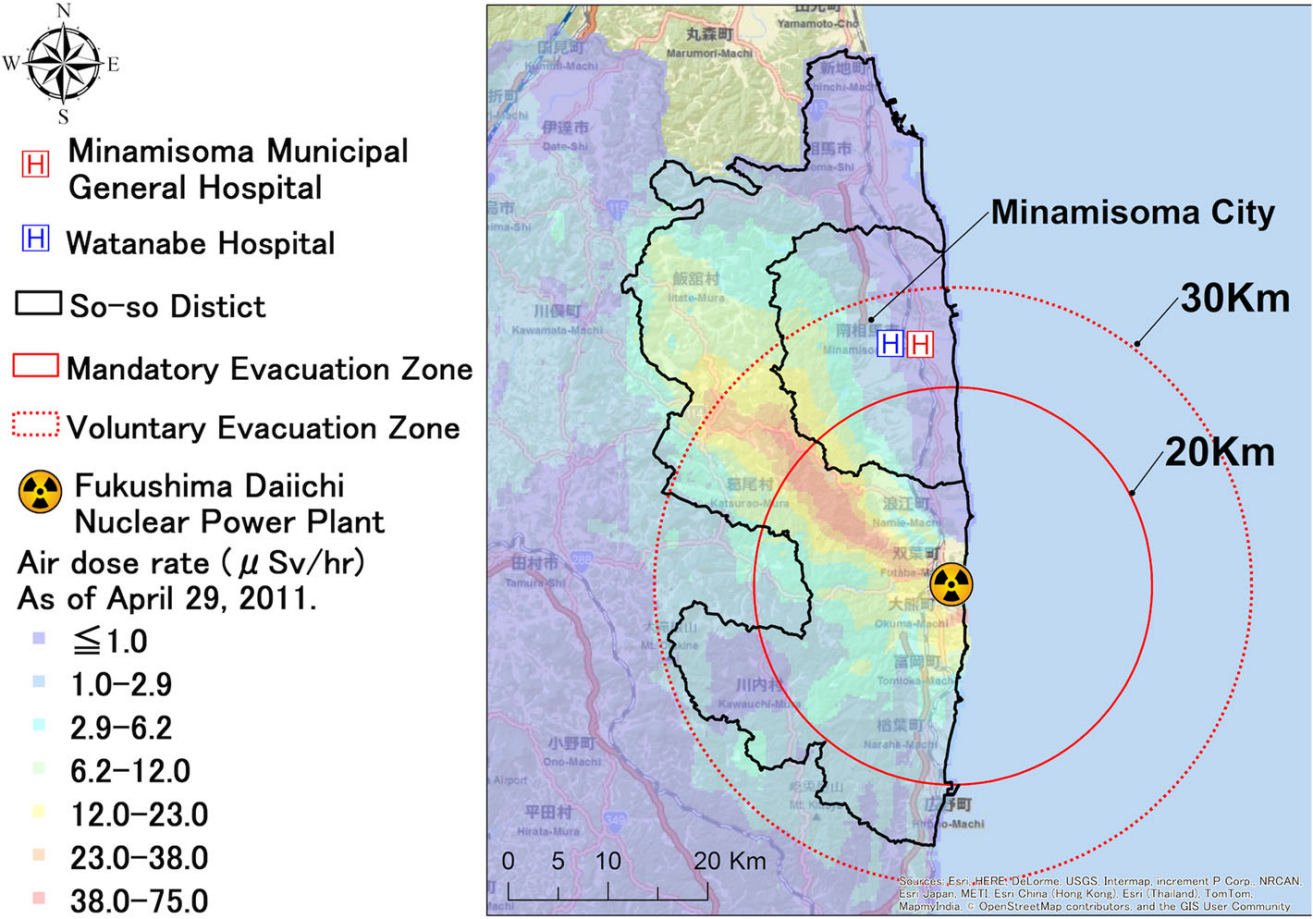
Map of Minamisoma City and its location within So-so District, Fukushima, with air dose rate. Minamisoma Municipal General Hospital and Watanabe Hospital are located 23 km and 25 km north of Fukushima Daiichi Nuclear Power Plant, respectively, both of which are within the voluntary evacuation zone. The air dose rate of radiation as of April 2011 is also described in this map. Approval for re-use of the image has been granted from ESRI Japan Corporation

On 11 March 2011, Japan experienced the Great East Japan Earthquake, ensuing tsunami, and the Fukushima Daiichi Nuclear Power Plant (FDNPP) disaster, referred to as Japan’s triple disaster [14, 15]. So-so District is located in the coastal area of Fukushima Prefecture, Japan, housing the FDNPP (Fig. 1), and was struck by all three disasters [16–19]. The central government issued mandatory evacuation orders for the 20 km radius around the nuclear power plant, and voluntary evacuation orders for the 20–30 km radius [11, 16, 19]. As a result, over 80,000 people in the mandatory evacuation zone were forced to evacuate [20], and more than 70,000 of them have continued evacuation as of 5 September 2015 [21]. In Minamisoma City, the largest municipality in So-so District, the original population of 72,000 drastically decreased to approximately 10,000 in April 2011, slowly recovering to 57,000 in October 2015 [16, 22]. As evacuation occurred primarily among young to middle-aged generations, the city has experienced rapid aging, with the proportion of elderly residents (≥ 65 years old) increasing from 26.5% in 2010 to 32.0% in 2015 [22]. Furthermore, the mean number of people per households has decreased from 3.00 in 2010 to 2.23 in 2015 [22]. Medical care services in the city were additionally affected, with the closure of 56% of medical institutions, and approximately a 15–20% decrease in health care providers in the two years post-disaster [16]. Moreover, fear of radiation exposure has persisted among local residents in Minamisoma compared to other areas of Fukushima [23], indicating the potential of long-lasting psychosocial stress amongst this population [24, 25]. These large-scale changes, including sociodemographic makeup of the city, access to healthcare, and psychosocial effects of the disaster are likely to have complicated cancer management and social support indispensable for cancer patients in the area, yet there is little information available on how breast cancer patients may have been affected to date.

The objectives of the present study are to identify 1) whether there was a post-disaster increase in the risk of experiencing patient delay among breast cancer patients, and 2) whether any of the following factors were associated with post-disaster patient delay: sociodemographic factors (age, cohabitant status and evacuation status), access to medical institutions, and psychosocial stress. The results of these inquiries will provide new information on the influence of mass disasters on patient delay.

## Methods

### Study settings and participants

This study took place at Minamisoma Municipal General Hospital (MMGH) and Watanabe Hospital (WH), located in Minamisoma City (Fig. 1). MMGH and WH stopped outpatient services immediately after the disaster, yet both restarted these services in June 2011. WH was the only medical institution in So-so District with an attending physician specialized in breast cancer care before the disaster. However, it lost inpatient functioning post-disaster, and stopped surgical therapy and chemotherapy for breast cancer patients. In August 2011, the WH breast cancer specialist moved to MMGH to restart breast cancer care for local residents, and since then, MMGH has been the only medical institution with a breast cancer specialist in So-so District (as of July 2016). These two hospitals are therefore considered to be the core breast cancer centers of So-so District.

To evaluate the impact of the 3.11 triple disaster on patient delay among breast cancer patients in So-so District, we retrospectively assessed the records of symptomatic breast cancer patients, newly diagnosed based on pathological findings, with first presentation to either of MMGH or WH from 1 January 2005 to 10 March 2016. The study period of 2005–2016 was chosen in order to assess any long-term influence of the disasters. Because this study focused on the period before a confirmatory diagnosis, we included patients who were later referred to other academic institutions or cancer centers after a pathological diagnosis of breast cancer in the two study hospitals. Patients with recurrent breast cancer, male patients, and those from outside So-so District were excluded. We also excluded those who were referred to MMGH or WH in the study period after first medical consultation to other medical providers during the non-study period. In the post-disaster period, we excluded those who had moved into the area following the disaster. Through the above process, patients were identified and categorized into two groups: pre- (from 1 January 2005 to 10 March 2011) or post-disaster (from 11 March 2011 to 10 March 2016).

### Analytical data

First, in order to assess the existence of patient delay, the date of first symptom discovery by patients or their families, and the date of the first medical consultation were collected from patient records. When a patient was referred from other medical providers, the first visit at the initial medical institution was considered as the first medical consultation.

Second, factors potentially associated with patient delay were extracted from the records. Sociodemographic factors at the first presentation were considered as follows: age [7, 8], cohabitant status (number of cohabitant family members, living with children or not, and living with a partner or not) [4, 8] and employment status (full-time job or not) [4, 8, 9]. Clinical characteristics were additionally included as follows; major symptoms (lump or non-lump) [9], hormone receptor (HR) status (positive or negative), stage at the diagnosis [5], American Society of Anesthesiologists (ASA) physical classification system (normal healthy patient, patient with mild systemic disease, and patient with severe systemic disease) [5, 26], body mass index (BMI) (<25 kg/m^2^, 25–30 kg/m^2^, or ≥30 kg/m^2^) [5], history of benign breast disease (yes or no) [8, 9], history of breast cancer (yes or no), mammography screening within two years (yes or no) [5], and family history of any kind of cancer (yes or no) [27, 28]. It has been suggested that HR-positive breast cancer progresses more slowly compared to HR-negative cancer [29]. Additionally considering that slow-developing cancers can lead to delayed medical consultation [30], HR status may influence length of patient interval. HR status was defined to be positive if either estrogen receptor status or progesterone receptor status was positive according to a cut-off value of no less than 1% in immunohistochemical (IHC) analysis [31]. As a population-based breast cancer screening is conducted through biennial mammography in Japan [32], previous experience of screening was classified according to whether there was attendance of mammography within the past two years. The details of family history of cancer, including which relatives had cancer and what kind of cancer they had, could not be examined because of data limitations. Four variables related to health care access were taken into account [3, 4]: hospital of the first medical consultation (MMGH or WH) and its linear distance to each patient’s residence, a referral to MMGH or WH from other medical providers (yes or no), and the interval from first medical consultation to first breast cancer specific examination (i.e. mammography, ultrasonography, or biopsy) [33].

Lastly, factors possibly associated with patient delay in disaster settings were gathered from patient records. In general settings, psychosocial distress can lead to a longer delay in first medical consultation among breast cancer patients [34]. A recent study suggested that evacuation status and residential air dose rates may be associated with psychosocial stress of local residents in the aftermath of Fukushima nuclear disaster [35]. We therefore considered it valuable to examine residential address of each patient and the air dose rate of radiation at their post-disaster residence in exploring the potential influence of psychosocial stress on post-disaster patient delay. The residential address of each patient was categorized into mandatory evacuation zone, voluntary evacuation zone, or non-evacuation zone of So-so District. For pre-disaster patients, residential addresses at the time of medical consultation were extracted, and for post-disaster patients, residential address at the time of the disaster was extracted. Methods of identifying the air dose rate for each participant’s residence are summarized in the next section. Further, medical costs of post-disaster patients were classified as free or not, according to the original addresses of participants; although Japan has universal health care [36], those who originally lived in mandatory evacuation zone or voluntary evacuation zone of Fukushima are now completely exempt from paying any medical fees as a disaster relief measure by the central government [37]. Hypothesized relationships between patient delay and factors studied are summarized in Table 1.

**Table 1:** Factors possibly associated with patient delay

Characteristics	Contributing factors / Protective factors
1. Sociodemographic factors	
Age	Contributing
Cohabitance with family	Protective
– Living with a partner	
– Living with children	
Full-time job	Contributing
2. Clinical factors	
Symptom of lump	Protective
Past history of benign breast disease	Protective
Past history of breast cancer	Protective
Family history of breast cancer	Protective
Underlying diseases	Contributing
Obesity	Contributing
Slowly developing cancer	Contributing
– Positive HR status	
Recent experience of mammography	Contributing
3. Factors representing health care access	
Adequate access to health service	Protective
– Short distance	
– Little or no cost	
– Sufficient medical providers	
– Short consultation interval	
4. Disaster-related factors	
Psychosocial stress	Contributing
– Evacuation status	
– Air dose rate	

### Measure of patient interval and patient delay

Patient interval was defined as the period from first recognition of a breast cancer-related symptom to the first medical consultation. According to the most generally used definition of patient delay [3, 6], total patient delay was defined as an interval of three months or longer. In order to assess patient delay comprehensively, we also introduced an additional category; excessive patient delay, defined as an interval of twelve months or longer [4, 38, 39].

### Air dose rate at home

After the FDNPP disaster, the Japanese Ministry of Education, Culture, Sports, Science, and Technology (MEXT) has conducted airborne radiation monitoring inside the 80 km radius of the FDNPP. The methods of these measurements have been documented in detail in a previous study [40]. All monitored results are publicly available [41].

In the present study, the post-disaster air dose rate at each participant’s residence was calculated using the following approach. We considered the results of the fourth MEXT monitoring performed between 22 October and 5 November 2011; sixth monitoring between 31 October and 16 November 2012; eighth monitoring between 2 and 19 November 2013; ninth between 1 September and 7 November 2014; and tenth between 12 September and 4 November 2015, for the radiation levels of the first (2011–2012), second (2012–2013), third (2013–2014), forth (2014–2015), and fifth (2015–2016) year post-disaster, respectively [41]. These values were averaged by a 500-m^2^ mesh on the basis of the Japan Profile for Geographical Information Standards (elevation and slope angle fourth mesh data) [42], and each participant’s house was assigned to a mesh area. This approach enabled estimation of the air dose rate at the home of each participant. In cases where the patients had experienced evacuation, post-evacuation addresses were used.

### Data analysis

We conducted two primary analyses. First, changes in the risk of experiencing total and excessive delay pre- and post-disaster were evaluated in the following manner. The overall and annual post-disaster incidence rates were respectively calculated by dividing the number of those with delays by the total number of breast cancer patients during each period from the date of the Great East Japan Earthquake, 11 March 2011 to 10 March 2016. For comparison, we calculated the overall incidence during pre-disaster period (from 1 January 2005 to 10 March 2011) by the same method, and utilized this value as a pre-disaster baseline. Based on these data, changes in risk in the overall post-disaster period (2011–2016) and each year post-disaster (2011–2012; 2012–2013; 2013–2014; 2014–2015; 2015–2016) compared to the pre-disaster baseline were identified as a risk ratio (RR), adjusted for age. Second, to identify factors associated with post-disaster patient delay, we constructed logistic regression models for total and excessive patient delay in post-disaster patients. Regression models for pre-disaster patient delay were also constructed, in order to address potential changes in the associations of factors with the patient delay pre- and post-disaster. All factors mentioned in the analytical data section were considered for this analysis. A *P* value of <0.05 was regarded as significant. All analyses were conducted by STATA/MP 14.1.

### Ethical approval

The ethics committee of MMGH assessed and granted approval for this study (approval number: 27–03). The ethics committee agreed that written consent from the participants was not required as this study was a retrospective analysis of patient records. All data were anonymized prior to analysis.

## Results

### Patient characteristics

**Fig. 2:**
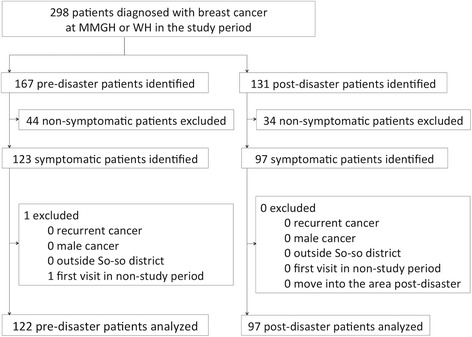
Process of participant inclusion

In the study period, 298 patients were newly diagnosed with breast cancer; 167 pre-disaster patients and 131 post-disaster patients. All non-symptomatic patients, comprised of 44 pre-disaster patients and 34 post-disaster patients, whose cancers were identified by a breast cancer examination (i.e. physical examination, mammography or ultrasonography) or incidentally, were excluded. Resultantly, there were 220 symptomatic patients in total; 123 pre-disaster patients and 97 post-disaster patients. Pre-determined exclusion criteria were then applied. One patient with a first consultation before the study period was excluded from the pre-disaster population. No patients with recurrent cancer, male patients, or those from outside So-so District were observed pre- or post-disaster. Additionally, no post-disaster patients had moved into the area following the disaster. There were 4 patients with a history of breast cancer defined as metachronal contralateral breast cancer cases, rather than recurrent cases. In total, 219 female breast cancer patients (73.5% of all cases, 99.5% of all symptomatic cases) were included, with 122 pre-disaster patients (73.1% of all pre-disaster cases, 99.2% of all pre-disaster symptomatic cases) and 97 post-disaster patients (74.0% of all post-disaster cases, 100.0% of all post-disaster symptomatic cases). The process of patient selection is displayed in Fig. 2.

Table 2 shows characteristics of the patients included in the analysis. There was no significant difference in the age distribution between pre- and post-disaster patients (Chi-squared test, *p* = 0.74). Additionally, distributions of breast cancer stage were not significantly different pre- and post-disaster (Fisher’s exact test, *p* = 0.22). However, a significantly smaller median number of cohabitant family members (1 vs. 2, *p* < 0.05), and a significantly smaller proportion of those living with children (47.4% vs. 59.0%, *p* < 0.05) were observed among post-disaster patients compared to pre-disaster patients. As number of cohabitant family member did not follow a normal distribution, we reported a median for this variable instead of its mean. With respect to clinical characteristics, there was no significant difference in the proportions of patients presenting with a lump between pre- and post-disaster patients (86.9% vs. 93.8%, *p* = 0.09). Regarding access to health service, there was no significant difference in the median distance between patient residence and study institutions at the first presentation (3.6 km vs. 3.3 km, *p* = 0.68) and the median days from first medical consultation to first breast cancer specific examination (0 vs. 0, *p* = 0.71) pre- and post-disaster. In the post-disaster period, there was a smaller proportion of patients who had resided in areas which became classified as parts of the mandatory evacuation zone, compared to the proportions of pre-disaster patients living in these areas (12.4% vs. 31.2%, *p* < 0.01). Also, in the post-disaster group, 84.5% of the patients were exempt from paying medical fees, and a mean air dose rate at patient’s residence was 0.31 μSv/h.

**Table 2:** Participants’ characteristics

Characteristic	Pre-disaster (*N* = 122)	Post-disaster (*N* = 97)	*P*-value
Age (N, %)			0.74
–50]	25 (20.5)	16 (16.5)	
(50–65]	41 (33.6)	33 (34.0)	
(65–	56 (45.9)	48 (49.5)	
Engaged in a full-time job (N, %)	24 (19.7)	22 (22.7)	0.59
Number of cohabitant family members^a^ (median, range)	2 (0–7)	1 (0–6)	<0.05
Living with a partner (N, %)			0.23
Yes	66 (54.1)	63 (65.0)	
No	50 (41.0)	34 (35.1)	
Missing	6 (4.9)	0 (0.0)	
Living with children (N, %)			<0.05
Yes	72 (59.0)	46 (47.4)	
No	44 (36.1)	51 (52.6)	
Missing	6 (4.9)	0 (0.0)	
Presence of lump (N, %)	106 (86.9)	91 (93.8)	0.09
Hormone receptor (N, %)			<0.001
Positive	80 (65.6)	89 (91.8)	
Negative	37 (30.3)	8 (8.3)	
Missing	5 (4.1)	0 (0.0)	
Stage (N, %)			0.22
0	11 (9.0)	5 (5.2)	
I	33 (27.1)	29 (29.9)	
II	48 (39.3)	42 (43.3)	
III	27 (22.1)	14 (14.4)	
IV	3 (2.5)	7 (7.2)	
ASA Physical classification system (N, %)			0.52
Normal healthy patient	59 (48.4)	40 (41.2)	
Patient with mild systemic disease	50 (41.0)	47 (48.5)	
Patient with severe systemic disease	13 (10.7)	10 (10.3)	
Body mass index (kg/m^2^, N, %)			0.33
–25]	75 (61.5)	62 (63.9)	
(25–30]	29 (23.8)	28 (28.9)	
(30–	14 (11.5)	6 (6.2)	
Missing	4 (3.3)	1 (1.0)	
History of benign breast disease (N, %)	7 (5.7)	6 (6.2)	0.89
History of breast cancer (N, %)	2 (1.6)	2 (2.1)	1.00
Undertook mammography screening within past two years (N, %)	15 (12.3)	12 (12.4)	0.99
Family history of any cancer (N, %)			0.46
Yes	50 (41.0)	46 (47.4)	
No	68 (55.7)	51 (52.6)	
Missing	4 (3.3)	0 (0.0)	
Hospital (N, %)			<0.001
Minamisoma Municipal General Hospital	14 (11.5)	97 (100)	
Watanabe Hospital	108 (88.5)	0 (0.0)	
Distance from hospital (median (km), range)	3.6 (0.2–47.0)	3.3 (0.2–22.8)	0.68
Referral from other medical providers (N, %)	55 (45.1)	33 (34.0)	0.10
Days from first medical consultation to first examination^b^ (median, range)	0 (0–1096)	0 (0–676)	0.71
Residential area^c^ (N, %)			<0.01
Non-evacuation zone of So-so District	13 (10.7)	15 (15.5)	
Voluntary evacuation zone	71 (58.2)	70 (72.2)	
Mandatory evacuation zone	38 (31.2)	12 (12.4)	
Exempt from medical fees^d^ (N, %)	N/A	82 (84.5)	
Air dose rate^de^ (average (μSv/h), age)	N/A	0.31 (0.17)	

^a^Data is missing in five pre-disaster patients

^b^Data is missing in one post-disaster patient

^c^For pre-disaster patients, this indicates place of residence at the time of first medical consultation, and for post-disaster patients, indicates place of residence at the time of Japan’s triple disaster

^d^Data is available only among post-disaster patients

^e^This indicates approximate air dose rate at home at the time of first medical consultation

### Delay in the first medical consultation

RRs of experiencing patient delay post- vs. pre-disaster are shown in Table 3. When we compared the overall post-disaster population with the pre-disaster baseline, there was a significant increase in the age-adjusted RR for both total patient delay (RR: 1.66, 95% Confidence Interval (CI): 1.02–2.70, *p* < 0.05) and excessive patient delay (RR: 4.49, 95% CI: 1.73–11.65, *p* < 0.01). According to the analysis of respective year, the RR of experiencing total patient delay peaked in the fourth year post-disaster (RR: 2.05, 95% CI: 1.10–3.81, *p* < 0.05), and that of excessive patient delay exceeded 5.0 in the second (RR: 5.58, 95% CI: 1.77–17.56, *p* < 0.01), fourth (RR: 5.27, 95% CI: 1.73–16.03, *p* < 0.01), and fifth year (RR: 5.24, 95% CI: 1.64–16.78, *p* < 0.01) post-disaster.

**Table 3:** Proportions and crude and age-adjusted risk ratios of patient delay post- versus pre-disaster (95% CI)

Characteristics	Proportion (%)	Crude risk ratio	Age-adjusted risk ratio
Total delay (≥3 months)			
Pre-disaster	18.0% (22/122)	Ref.	Ref.
Post-disaster			
Overall population	29.9% (29/97)	1.66 (1.02–2.69)*	1.66 (1.02–2.70)*
2011–2012^a^	20.0% (2/10)	1.11 (0.30–4.05)	1.11 (0.30–4.04)
2012–2013^a^	27.3% (6/22)	1.51 (0.69–3.30)	1.51 (0.69–3.30)
2013–2014^a^	26.7% (4/15)	1.48 (0.59–3.71)	1.49 (0.59–3.74)
2014–2015^a^	37.0% (10/27)	2.05 (1.10–3.82)*	2.05 (1.10–3.81)*
2015–2016^a^	30.4% (7/23)	1.69 (0.82–3.48)	1.75 (0.84–3.63)
Excessive delay (≥12 months)			
Pre-disaster	4.1% (5/122)	Ref.	Ref.
Post-disaster			
Overall population	18.6% (18/97)	4.53 (1.74–11.76)**	4.49 (1.73–11.65)**
2011–2012^a^	10.0% (1/10)	2.44 (0.31–18.91)	2.44 (0.31–18.85)
2012–2013^a^	22.7% (5/22)	5.55 (1.75–17.57)**	5.58 (1.77–17.56)**
2013–2014^a^	6.7% (1/15)	1.63 (0.20–13.01)	1.63 (0.20–12.94)
2014–2015^a^	22.2% (6/27)	5.42 (1.78–16.47)**	5.27 (1.73–16.03)**
2015–2016^a^	21.7% (5/23)	5.30 (1.67–16.87)**	5.24 (1.64–16.78)**

^a^In each period, the starting date is 11 March

*<0.05, **<0.01

Patient interval, instead of date of first symptom recognition, was reported in the majority of patient records. In such cases, the reported interval was directly collected from the patient records. We summarize sociodemographic factors, patient interval and date of first presentation for patients with excessive patient delay for both pre- and post-disaster in Table 4. Among the 18 patients with excessive patient delay in the post-disaster group, 27.8% (5/18) discovered their symptoms before the disaster.

**Table 4:** Profiles of patients with excessive patient delay pre- and post-disaster

Patient	Age	First presentation	Patient interval	Residential area^a^	Full-time job	Cohabitant children
Pre-disaster						
1	(50–65]	Apr.-Jun. 2007	120 months	Voluntary evacuation zone	Yes	No
2	(65-	Apr.-Jun. 2008	48 months	Voluntary evacuation zone	No	No
3	(50–65]	Sep.-Nov. 2008	18 months	Voluntary evacuation zone	No	No
4	(65-	Dec. 2008-Feb. 2009	120 months	Non-evacuation zone	No	Yes
5	−50]	Sep.-Nov. 2009	36 months	Mandatory evacuation zone	Yes	Yes
Post-disaster						
1	(50–65]	Dec. 2011-Feb. 2012	24 months	Non-evacuation zone	No	Yes
2	(65-	Jun.-Aug. 2012	18 months	Non-evacuation zone	No	No
3	(50–65]	Sep.-Nov. 2012	17 months	Voluntary evacuation zone	Yes	No
4	(65-	Sep.-Nov. 2012	12 months	Voluntary evacuation zone	No	No
5	−50]	Dec. 2012-Feb. 2013	12 months	Voluntary evacuation zone	No	No
6	(65-	Dec. 2012-Feb. 2013	25 months	Voluntary evacuation zone	Yes	No
7	(65-	Mar.-May 2013	24 months	Voluntary evacuation zone	No	No
8	(65-	Mar.-May 2014	12 months	Non-evacuation zone	No	No
9	−50]	Jun.-Aug. 2014	13 months	Non-evacuation zone	Yes	Yes
10	(65-	Sep.-Nov. 2014	24 months	Voluntary evacuation zone	No	No
11	(65-	Sep.-Nov. 2014	84 months	Voluntary evacuation zone	No	Yes
12	−50]	Sep.-Nov. 2014	24 months	Voluntary evacuation zone	No	No
13	(65-	Dec. 2014-Feb. 2015	24 months	Voluntary evacuation zone	No	No
14	(50–65]	Jun.-Aug. 2015	24 months	Mandatory evacuation zone	No	No
15	(65-	Sep.-Nov. 2015	24 months	Voluntary evacuation zone	No	No
16	(65-	Sep.-Nov. 2015	120 months	Voluntary evacuation zone	No	No
17	(50–65]	Sep.-Nov. 2015	13 months	Mandatory evacuation zone	No	Yes
18	(50–65]	Dec. 2015-Feb. 2016	12 months	Voluntary evacuation zone	Yes	No

^a^For pre-disaster patients, this indicates place of residence at the time of first medical consultation, and for post-disaster patients, indicates place of residence at the time of Japan’s triple disaster

### Factors related to post-disaster patient delay

The results of univariate logistic regression analysis for pre- and post-disaster total patient delay are shown in Tables 5 and 6, respectively. In the pre-disaster period, no associations between total patient delay and access-related factors, sociodemographic factors, or other clinical characteristics studied were observed. In the post-disaster period, none of access- and disaster-related factors and sociodemographic factors were significantly associated with experiencing total patient delay, however a significant association was observed with having a family history of any cancer (odds ratio: 0.38, 95% CI: 0.15–0.95, *p* < 0.50). Due to lack of significant variables, we did not conduct multivariate logistic regression analysis for total patient delay.

**Table 5:** Pre-disaster odds ratios for patient delay (univariate regressions)

Variable	Odds Ratio (95% CI)	Patients analyzed (No.)
Hospital		122
Watanabe Hospital	Ref.	108
Minamisoma Municipal General Hospital	1.28 (0.32–5.02)	14
Distance from hospital	1.02 (0.96–1.08)	122
Referral from other medical providers		122
No	Ref.	67
Yes	1.99 (0.78–5.10)	55
Days from first medical consultation to first examination	1.00 (0.98–1.02)	122
Residential area^a^		122
Non-evacuation zone of So-so District	Ref.	13
Voluntary evacuation zone	2.69 (0.32–22.56)	71
Mandatory evacuation zone	3.20 (0.36–28.42)	38
Age		122
–50]	Ref.	25
(50–65]	0.82 (0.23–2.94)	41
(65–	0.87 (0.26–2.87)	56
Engaged in a full-time job		122
No	Ref.	98
Yes	1.25 (0.41–3.82)	24
Number of cohabitant family members		117
0–1	Ref.	44
2–3	1.95 (0.58–6.55)	40
More than 4	2.50 (0.73–8.50)	33
Living with a partner		116
No	Ref.	50
Yes	1.01 (0.39–2.63)	66
Living with children		116
No	Ref.	44
Yes	0.99 (0.37–2.62)	72
Lump		122
No	Ref.	16
Yes	0.95 (0.25–3.65)	106
Hormone receptor		117
Negative	Ref.	37
Positive	1.19 (0.42–3.37)	80
Stage		122
0	Ref.	11
1	2.22 (0.24–20.83)	33
2	1.71 (0.19–15.51)	48
3 or 4	3.64 (0.40–33.12)	30
ASA physical classification system		122
Normal healthy patient	Ref.	59
Patient with mild systemic disease	1.39 (0.52–3.74)	50
Patient with severe systemic disease	1.67 (0.38–7.27)	13
Body mass index (kg/m^2^)		118
–25]	Ref.	75
(25–30]	1.37 (0.46–4.07)	29
(30–	2.10 (0.56–7.81)	14
History of benign breast disease		122
No	Ref.	115
Yes	0.75 (0.09–6.53)	7
Undertook mammography screening within past two years		122
No	Ref.	107
Yes	2.65 (0.80–8.72)	15
Family history of any cancer		118
No	Ref.	68
Yes	1.64 (0.63–4.22)	50

^a^This indicates place of residence at the time of first medical consultation

**Table 6:** Post-disaster odds ratios for patient delay (univariate regressions)

Variable	Odds Ratio (95% CI)	Patients analyzed (No.)
Hospital^a^		97
Watanabe Hospital	N/A	0
Minamisoma Municipal General Hospital	N/A	97
Distance from hospital	0.96 (0.89–1.05)	97
Referral from other medical providers		97
No	Ref.	61
Yes	1.58 (0.64–3.89)	33
Days from first medical consultation to first examination	1.00 (1.00–1.01)	96
Residential area^b^		97
Non-evacuation zone of So-so District	Ref.	15
Voluntary evacuation zone	1.26 (0.36–4.40)	70
Mandatory evacuation zone	0.92 (0.16–5.21)	12
Age		97
–50]	Ref.	16
(50–65]	1.50 (0.39–5.75)	33
(65–	1.24 (0.34–4.49)	48
Engaged in a full-time job		97
No	Ref.	75
Yes	1.47 (0.54–4.01)	22
Number of cohabitant family members		97
0–1	Ref.	49
2–3	0.68 (0.26–1.77)	34
More than 4	0.51 (0.13–2.09)	14
Living with a partner		97
No	Ref.	34
Yes	1.29 (0.51–3.27)	63
Living with children		97
No	Ref.	51
Yes	0.58 (0.24–1.40)	46
Lump		97
No	Ref.	6
Yes	0.19 (0.03–1.10)	91
Hormone receptor		97
Positive	Ref.	89
Negative	0.69 (0.15–3.09)	8
Stage		92
0	N/A	5
1	N/A	29
2	N/A	42
3 or 4	N/A	21
ASA physical classification system		97
Normal healthy patient	Ref.	40
Patient with mild systemic disease	1.27 (0.49–3.29)	47
Patient with severe systemic disease	3.00 (0.72–12.55)	10
Body mass index (kg/m^2^)		96
–25]	Ref.	62
(25–30]	1.71 (0.67–4.39)	28
(30–	0.53 (0.06–4.87)	6
History of benign breast disease		97
No	Ref.	91
Yes	1.19 (0.20–6.86)	6
Undertook mammography screening within past two years		97
No	Ref.	85
Yes	0.76 (0.19–3.02)	12
Family history of any cancer		97
No	Ref.	51
Yes	0.38 (0.15–0.95)*	46
Air dose rate [μSv/h]	0.71 (0.05–10.03)	97

^a^Odds ratio was not calculated because Watanabe Hospital stopped inpatient oncology services post-disaster

^b^This indicates place of residence at the time of Japan’s triple disaster

*<0.05

We attempted to conduct logistic regression analyses for excessive patient delay in respective pre- and post-disaster periods. However, it was difficult to establish a stable model of pre-disaster excessive patient delay owing to limited numbers of participants with this delay. We present characteristics of pre- and post-disaster patients stratified by with or without excessive and total patient delay in Additional files 1 and 2. Only 22.2% (4/18) of post-disaster patients with excessive patient delay lived with their children, compared to 53.2% (42/79) of those without excessive patient delay.

## Discussion

In this long-term retrospective study of 219 patients with symptomatic breast cancer in an area severely damaged by the 2011 triple disaster, we found an increased risk of patient delay among post-disaster patients compared to those pre-disaster, a trend which has continued for five years after the disaster. Additionally, a smaller proportion of the patients living with children and lower median number of cohabitant family members were observed post-disaster, compared with the pre-disaster period. However, we could not elucidate contributing or protective factors for post-disaster patient delay, aside from a family history of cancer (Tables 5 and 6).

The extent of increased risk in both total and excessive patient delay after the disaster was prominent. Although the proportion of those with total patient delay was 18.0% pre-disaster, a similar range as that observed in routine clinical settings of high-income countries (HICs) [5, 43], it reached 29.9% post-disaster, a level compatible to that observed in low- and middle-income countries (LMICs) [3]. Furthermore, 18.6% of all post-disaster patients experienced excessive patient delay, compared to only 4.1% pre-disaster. Generally, the definition of excessive patient delay is adopted in studies conducted in LMICs [4, 6, 39], as this type of prolonged delay occurs more frequently in LMICs compared to HICs [3]. However, we hypothesize that irregular circumstances, such as disasters, could cause excessive patient delay even in HICs, as suggested by the results of this study. It is to be noted that possible reasons for patient delay in post-disaster Fukushima could be different from those in LMICs, where limited access to health care and poor knowledge or awareness of breast cancer are regarded as the primary factors contributing to patient delay [3, 4]. The potential contributing factors to patient delay in our study are discussed in the following sections, including health care access, social support from family members, and psychosocial stress.

The present findings are in line with previous studies which showed a devastating impact of mass disasters on general aspects of cancer management [12, 13, 44–46]. Yet, while these reports primarily focused on the immediate aftermath of disasters [12, 13, 44, 45], our study revealed a long-lasting effect of the triple disaster on those with breast cancer in affected areas. Moreover, although immediate deterioration in healthcare access has been underlined in previous studies [13, 44, 46], breast cancer oncology services were essentially recovered and maintained in So-so District from three months post-disaster. Indeed, healthcare access, measured as linear distance between one’s residence and hospital of first medical consultation (MMGH or WH), referral from other medical providers, or interval from first medical consultation to first breast cancer specific examination, did not differ significantly pre- and post-disaster despite closure of oncology service in WH. Furthermore, no associations were found between these variables and total patient delay. It is additionally to be noted that Minamisoma City has continuously provided mammography screening to local residents throughout the post-disaster period. It can therefore be argued that, rather than changes in healthcare access, alternative mechanisms may have contributed to patient delay among post-disaster breast cancer patients in the present study.

It is notable that there was a significant decrease in proportion of those living with children (47.4% vs. 59.0%, *p* < 0.05) and the median number of cohabitant family members (1 vs. 2, *p* < 0.05) post-disaster compared with pre-disaster. There findings are compatible with demographic changes that Minamisoma City has experienced post-disaster [22]. It is true that all regression results regarding factors related to cohabitant status were null (Tables 5 and 6). However, it can be speculated that these post-disaster demographic changes may have contributed to decreased social support patients could acquire from family members, increasing the risk of experiencing patient delay among this population. In fact, previous studies have demonstrated the importance of social support to the process of seeking medical attention in disaster settings [13, 47, 48]. In particular, social support from children, rather than partners, may lessen the risk of patient delay and improve the prognosis of breast cancer patients [7, 49]. In the post-disaster period, only 22.2% (4/18) of patients with excessive patient delay lived with their children, compared to 53.2% (42/79) of patients without excessive patient delay (Additional file 1), further supporting our hypothesized relationship between poor social support and patient delay after the disaster.

We could not find any associations between post-disaster patient delay and variables, such as evacuation status and air dose rate at residence. We speculated that these variables could reflect post-disaster psychosocial stress among breast cancer patients, because they were found to be associated with psychosocial stress in Fukushima residents in a previous study [35]. However, it is possible that psychosocial stress may not have been captured accurately by these markers in the present study. Obtaining reliable information on radiation risks, such as environmental radiation level at one’s residence, may have been challenging for local residents, as repeatedly suggested in previous studies [11, 25, 50]. Resultantly, It is possible that local residents of Minamisoma City may feel anxiety over potential radiation exposure, regardless of individual measurement results in their area [23, 51]. Additionally, there have been several hypotheses made for sources of psychosocial stressors after the Fukushima nuclear incident, apart from radiation exposure, as follows; decades of work expected to be necessary for decommissioning the FNDPP reactor [52], discordant perceptions of radiation risks among families and communities, and community tensions which have occurred as a result of disparities in governmental compensations and restrictions [25, 53]. In order to comprehensively evaluate any relationships between psychosocial stress and patient delay after this disaster, a qualitative method may be useful, as its efficacy has been demonstrated in multiple previous studies on breast cancer patient delay in general settings [2, 4, 8].

There was a significantly higher proportion of HR-positive breast cancer after the disaster, compared with the pre-disaster period (91.8% vs. 65.6%, *p* < 0.001). Breast cancer can be affected by radiation exposure [54], and its incidence may increase 10–15 years following serious radiation exposure [54], as seen in the areas seriously contaminated after the Chernobyl nuclear power plant accident [55]. However, the World Health Organization and the United Nations Scientific Committee on the Effects of Atomic Radiation have recently concluded that lifetime risk of developing cancer is marginal among adults in the general public exposed to the Fukushima nuclear disaster [56, 57]. Therefore, it appears unlikely that radiation exposure would have caused the increased proportions of HR positivity in the present study. Notably, while 88.5% of pre-disaster patients first visited WH, all of the post-disaster patients visited MMGH. Taking into account the changes local breast cancer oncology service has experienced, discordance in interpreting and reproducing HR positivity among antibodies utilized in IHC analysis and pathologists may have contributed to the present findings [58, 59].

We find it possible that our present findings on patient delay may be applicable to other types of cancer in disaster settings, as social disruption and psychosocial stress among cancer patients may not be limited to breast cancer in such situations [13, 47]. Notably, studies performed in non-disaster settings have suggested that patients with breast cancer were least likely to delay their presentation compared to those with colorectal, urological, gynecological or hematological cancer [60], and that that social support and emotional health of cancer patients can widely influence how soon they make first medical consultations, regardless of cancer type [61–63]. It may be therefore reasonable to hypothesize that patients with other types of cancer may delay their first medical consultation to the same extent or more than breast cancer patients in disaster settings. We suggest that further studies should be conducted to assess applicability of the present results to other cancers.

### Limitations

This study has several limitations. First, it is possible that selection bias may have influenced our results. A potential effect of post-disaster demographic changes, primarily due to mass-evacuation among young- and middle-aged generations, should be acknowledged as it may have resulted in differences between the pre- and post-disaster populations of So-So district. For example, if younger residents evacuated and did not return, an older population may naturally have led to larger proportions of patient delay [7, 8], which could have led to an overestimation of our results. However, aside from differences in areas of residential address and cohabitant status, we could find no significant differences in demographics of pre- and post-disaster participants, and it is particularly notable that there were no changes in the age distribution of patients in pre- and post-disaster periods. Second, although we included as many breast cancer patients as possible in the affected areas based on the data from MMGH and WH, there was still a limited sample size that provided low power for analyses and the observed results may have been affected by random error. Third, in most cases, patient intervals were reported instead of date of symptom recognition. As patient intervals lengthened, these variables may be more easily affected by recall biases. Finally, we could not evaluate some factors known to be associated with patient delay but not reported in patient records, including breast cancer knowledge, personalities, and sources of social support apart from family members [4, 8, 49].

## Conclusions

This is the first study to assess the impact of the 2011 triple disaster on cancer patients in a severely affected area of Fukushima. The risk of experiencing patient delay was significantly higher post-disaster, compared to the pre-disaster period, and this trend has continued for five years following the disaster. This study presents unprecedented information on breast cancer patient delay in the long-term aftermath of a disaster.

## Additional files

Additional file 1: Patient characteristics of excessive patient delay pre- and post-disaster. (XLSX 13 kb)

Additional file 2: Patient characteristics of total patient delay pre- and post-disaster. (XLSX 13 kb)

## Abbreviations

ASA: American Society of Anesthesiologists
BMI: Body mass index
CI: Confidence Interval
FDNPP: Fukushima Daiichi Nuclear Power Plant
HICs: High-income counties
LMICs: Low- and middle-income countries
HR: Hormone receptor
IHC: Immunohistochemical
MEXT: Ministry of Education, Culture, Sports, Science, and Technology
MMGH: Minamisoma Municipal General Hospital
RR: Risk ratio
WH: Watanabe Hospital
